# Aural Myiasis: A Case Report on a Rare Entity

**DOI:** 10.7759/cureus.10617

**Published:** 2020-09-23

**Authors:** Ellen Rummens, Gerry Van der Mieren, Vincent Van Rompaey, Peter Piessens, Francis Somville

**Affiliations:** 1 Otolaryngology - Head and Neck Surgery, ASZ Aalst, Aalst, BEL; 2 Emergency Medicine, AZ Dimpna, Ziekenhuis Geel, Geel, BEL; 3 Otolaryngology - Head and Neck Surgery, University Hospital of Antwerp, Antwerpen, BEL; 4 Otolaryngology - Head and Neck Surgery, AZ Dimpna, Ziekenhuis Geel, Geel, BEL

**Keywords:** aural myiasis, ear infection, mastoid, myiasis infestation

## Abstract

Myiasis is the infestation of live vertebrates with dipterous larvae. It is a rare entity in the otolaryngology and is more common to occur in patients with mental or physical disabilities. There are only few cases reported in the literature, and most cases are seen in tropical and rural areas. In this case report, we present a 65-year-old patient, with a history of parotid malignancy, who presented with aural myiasis with extension to the mastoid. We discuss the clinical presentation, the further examinations, and the treatment for early- and late-stage infection.

## Introduction

Myiasis is the infestation of live vertebrates (humans and/or animals) with dipterous larvae. In mammals (including humans), dipterous larvae can feed on the host's living or dead tissue, liquid body substance, or ingested food and cause a broad range of infestations depending on the body location and the relationship of the larvae with the host [1]. Myiasis is a rare entity in otolaryngology. It can be found in the ears, nose and paranasal sinuses, nasopharynx, oral cavity, skin of the head and neck region, larynx, and trachea [1,2]. Aural myiasis, or otomyiasis, involves the infestation of the external ear and/or middle ear [1]. Predisposing factors are chronic suppurative otitis media, low socioeconomic status, swimming in stagnant water, diabetes mellitus, neglected children, old age, mental retardation, and poor personal hygiene [2]. Early intervention is necessary to avoid complications like deafness or intracranial extension [1,3]. Diagnosis is mostly made by history and clinical examination. Further examinations are necessary when expansion to the middle ear is suspected [2]. 

## Case presentation

A 65-year-old patient presented with purulent, occasionally haemorrhagic ear discharge, pruritus, pain, and a murmur in the right ear since a couple of days. There was no facial weakness. The patient did not have a fever and was alert. He had poor personal hygiene.

He had a history of an ipsilateral parotid malignancy treated with surgery, chemotherapy, and radiotherapy. Social history revealed that he used to be a wood worker, but retired seven years ago. He had no pets, nor made trips to exotic destinations.

**Figure 1:**
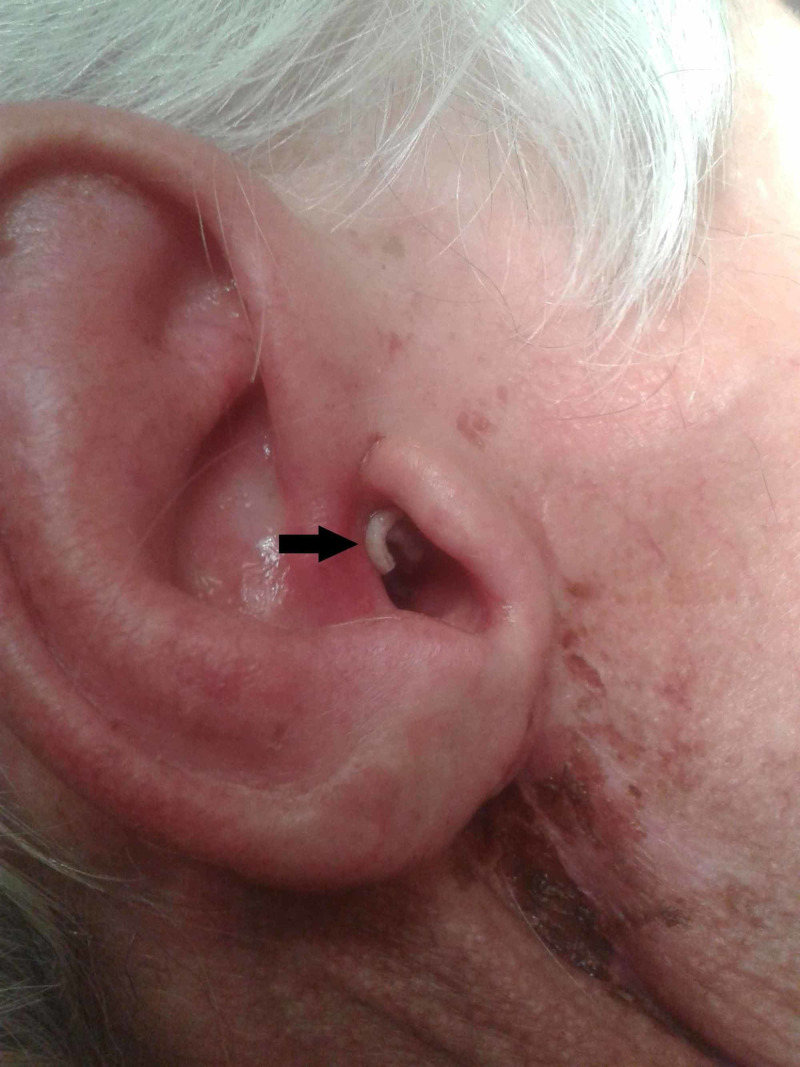
Mobile white foreign objects (black arrow) in the right ear of the patient.

**Figure 2:**
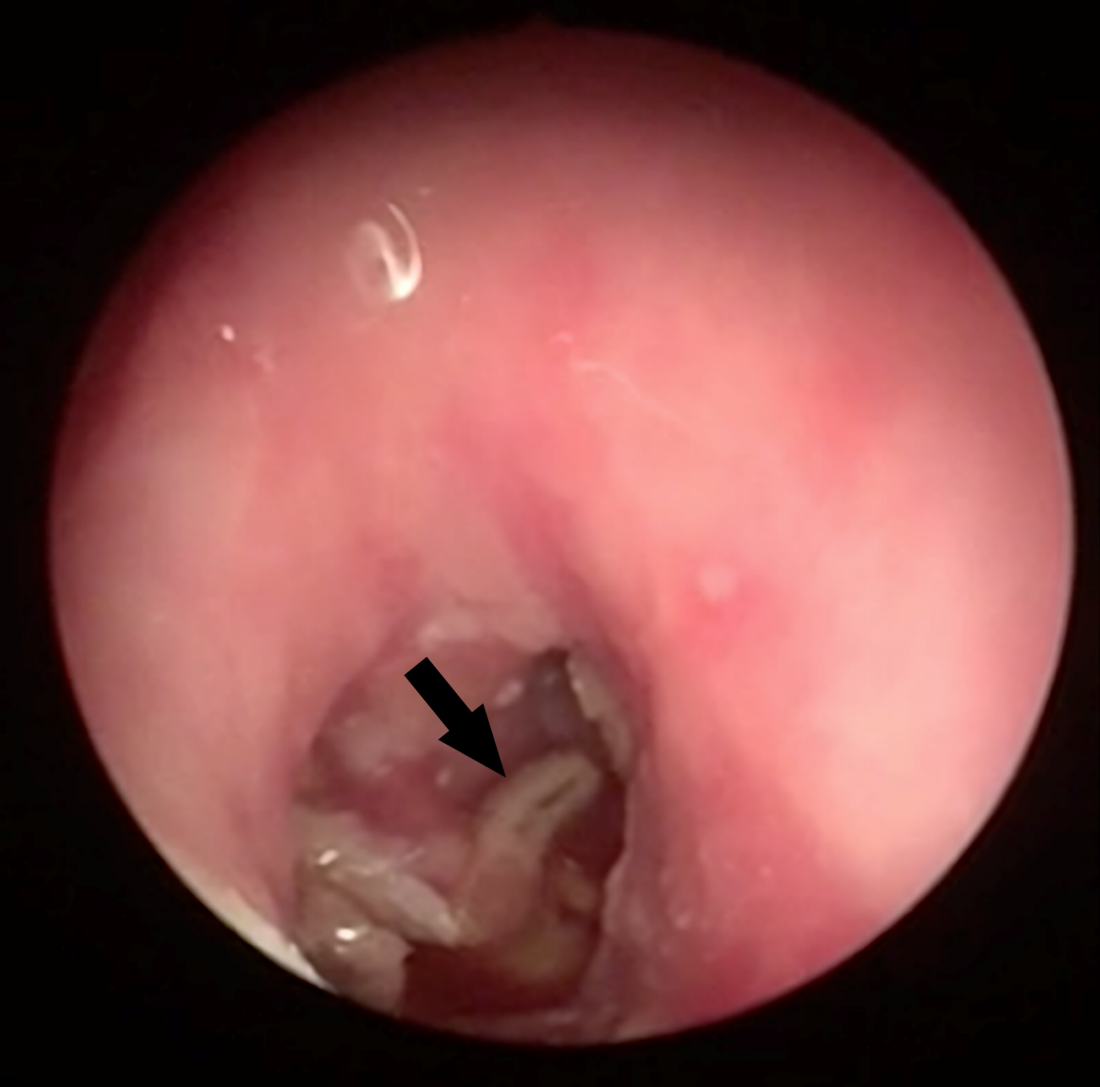
Endoscopic examination of the ear with presence of multiple maggots (black arrow), no identifiable tympanic membrane.

Clinical examination showed an erosive lesion behind the right ear with a scar of a previous parotidectomy. Upon a closer look, we observed several mobile white foreign objects (Figure 1) of which we acquired a specimen for further examination. Otoscopic examination showed oedema of the external auditory canal and maggots. There was no identifiable tympanic membrane. There were bony sequestrations and granulation tissue in the middle ear. During inspection of the mastoid, a fistula was observed connecting the skin to the mastoid cavity (Figure 2). The cranial nerves, with special attention to the facial nerve, were intact. Palpation of the neck did not reveal any cervical lymphadenopathies. Examination of the mouth did not show trismus but there was bad dental hygiene. There was a lack of overall hygiene. 

**Figure 3:**
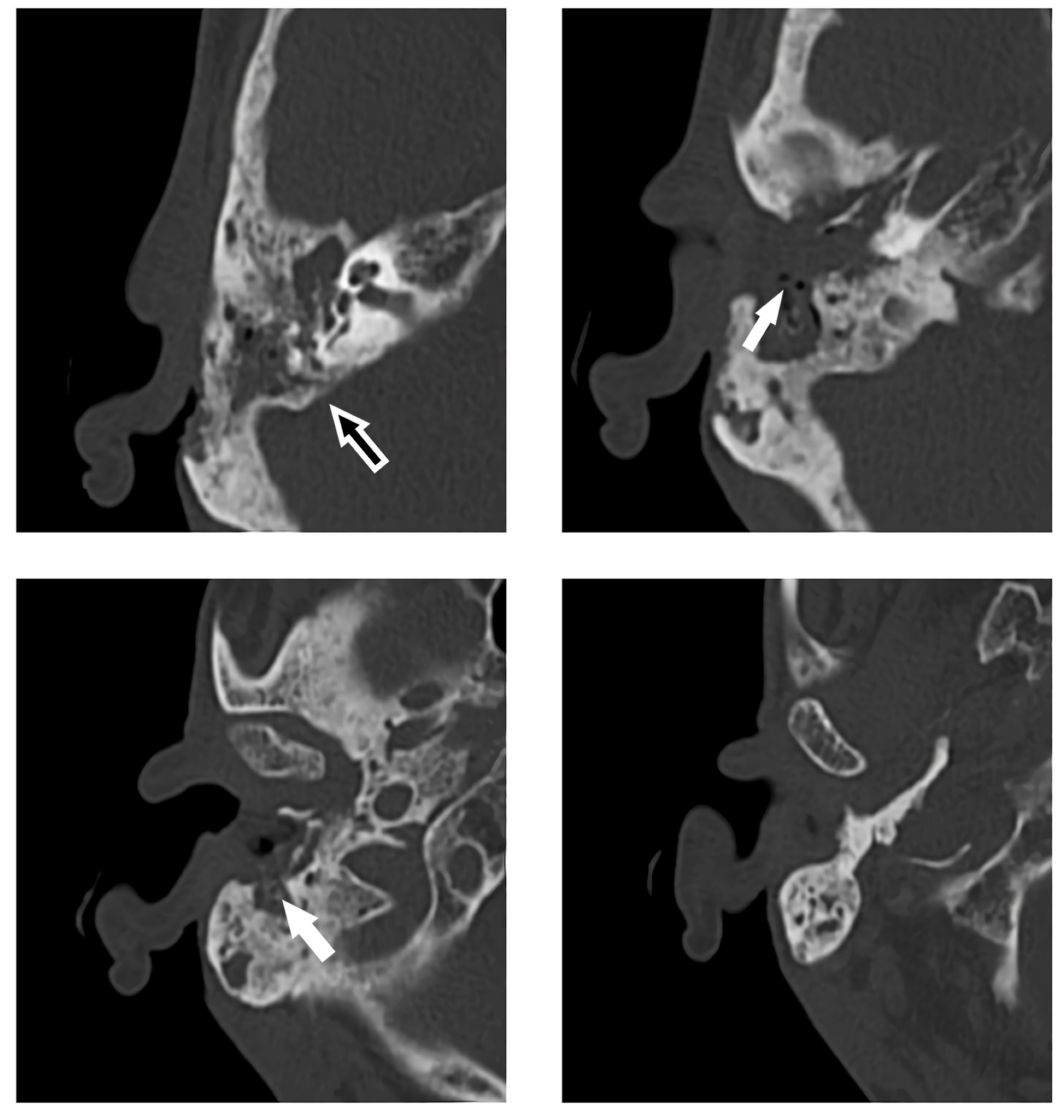
CT scan (axial plane) of mastoid with erosive infestation of the anterior (white arrow) and posterior (black arrow) bony wall of the external ear canal, focal dehiscence of the cranial wall of the mastoid, and opacification of the mastoid air cells and middle ear.

A CT scan showed erosive infestation of the anterior and posterior bony wall of the external ear canal, focal dehiscence of the cranial wall of the mastoid, and opacification of the mastoid air cells and middle ear (Figure 3).

A blood sample showed normal white blood cell count with a mild lymphocytopenia (17.2%, normal range 20%-45%), slightly increased D-dimers (593 ng/mL, normal range 0-549), and C-reactive protein (25 mg/L, normal range < 5). Liver function tests, serum electrolytes, urea, and creatinine were within normal range.

The specimen revealed human botfly or Dermatobia hominis.

**Figure 4:**
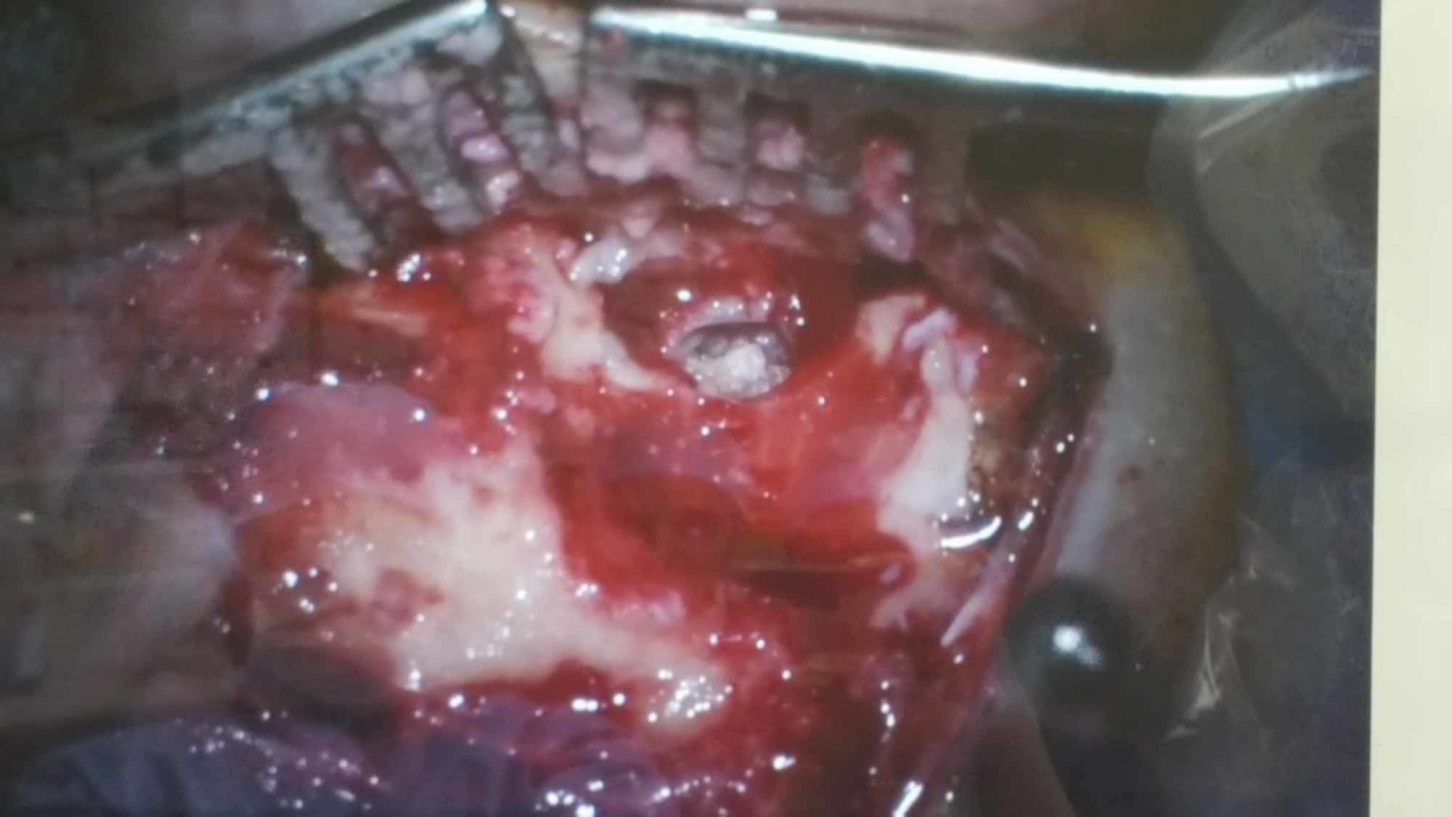
Surgical removal of the maggots and necrotic tissue and subtotal petrosectomy.

To avoid intracranial complications, an emergency subtotal petrosectomy was performed with resection of the larvae, necrotic bony sequesters, and affected mucosa. The incus and malleus were absent, and the stapes was mobile. The mastoid was obliterated using a pedicled temporal muscle flap (Figure 4). 

Postoperative treatment consisted of amoxicillin-clavulanic acid to prevent secondary infection and local treatment with a terramycine and hydrocortisone suspension. Four months after surgery, no recurrence was observed.

## Discussion

Myiasis is a relatively common public health problem in developing countries, mainly in hot, tropical areas. There are, however, no clinical practice guidelines for diagnosis or treatment of this disease. Otomyiasis occurs when the female fly deposits its larvae in the auditory canal [4].

Infection is more common in tropical locations with a warm and humid environment and manifestation commonly occurs in patients with poor personal hygiene, children, and mentally retarded adults [5,6]. Patients mostly present themselves with sensation of foreign substance in the ear, aural itching, pain, bleeding, tinnitus, hearing loss, and vertigo [7-9].

Myiasis is diagnosed by clinical examination and otomicroscopy, which may reveal maggots, an inflamed and swollen auditory canal, and in some cases, tympanic membrane perforation [3]. A CT scan can be performed to exclude extension of the infection to the mastoid or intracranial and is recommended before surgical exploration [3,4,10,11].^ ^

Treatment of the condition is necessary to avoid (intracranial) complications.

Early stages of aural myiasis can be treated with maintaining simple aural toilet with suction and topical and/or oral antibiotic coverage. Different substances can be used as treatment: chloroform, oil drops, urea, dextrose, keratin, hypertonic saline, and iodine solution [2,6,9,11-13]. The goal of the irrigation is to kill and expel any residual larvae solution. Therefore, the choice of the solution is debatable as all of them achieve the same outcome [2].

In cases of late-stage aural myiasis, extensive surgical debridement of the infected tissue and reconstruction is necessary [13]. In advanced cases with purulent otorrhoea or when involving deep cavities, systemic antibiotic treatment is recommended. When there is expansion to the middle ear through the tympanic membrane, when expansion to the brain is suspected, or in cases of residual disease, surgical management with mastoid exploration is advised [2,13]. Care should be taken to obliterate the mastoid cavity to exclude any cavity that can be a source of recurrence. In this case, a pedicled muscle flap was used because of the patient’s history of radiation therapy in the head and neck region.

## Conclusions

Aural myiasis is a rare but dangerous condition especially if the middle ear and mastoid bone are involved because of the possibility of penetration to the brain. The treatment in early manifestation consists of removal of the larvae and irrigation of the ear. Prophylactic broad-spectrum antibiotics are prescribed to prevent secondary infections. Surgical treatment is necessary when the disease extends beyond the auditory canal and/or tympanic membrane.
